# Dichlorido{(*E*)-2,4,6-trimethyl-*N*-[phen­yl(2-pyridyl)methyl­idene]aniline-κ^2^ *N*,*N*′}palladium(II)

**DOI:** 10.1107/S1600536810016466

**Published:** 2010-05-08

**Authors:** Cheng-Hsien Yang, Ya-Liu Peng, Mei-Hua Wang, Kuo-Chen Shih, Mao-Lin Hsueh

**Affiliations:** a Nano-Powder & Thin Film Technology Center, ITRI South, Tainan 709, Taiwan, Republic of China; b Department of Chemistry, National Chung Hsing University, Taichung 402, Taiwan, Republic of China

## Abstract

The title complex, [PdCl_2_(C_21_H_20_N_2_)], contains a Pd^II^ atom in a slightly distorted square-planar coordination environment defined by two N atoms from one 2,4,6-trimethyl-*N*-[phen­yl(2-pyrid­yl)methyl­idene]aniline ligand and two Cl atoms, forming a five-membered ring (N—Pd—N—C—C).

## Related literature

For the synthesis of pyridyl-imine ligands, see: Meneghetti *et al.* (1999 ▶). For the design and synthesis of metal-organic frameworks, see: Lai *et al.* (2005 ▶); Pelagattia *et al.* (2005 ▶); Zhang *et al.* (2008 ▶). For related structures, see: Hsueh *et al.* (2006 ▶); Zhang *et al.* (2008 ▶). For the application of the title compound in Suzuki–Miyaura reactions, see: Li (2003 ▶); Miyaura & Suzuki (1995 ▶); Na *et al.* (2004 ▶); Nicolaou *et al.* (2005 ▶); Rajagopal *et al.* (2002 ▶); Tomioka *et al.* (2004 ▶).
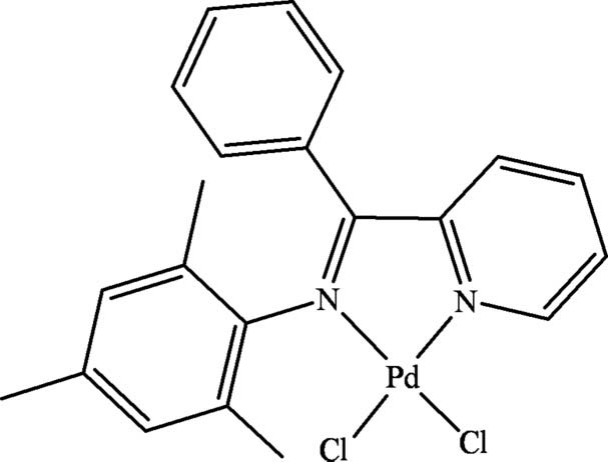


## Experimental

### 

#### Crystal data

[PdCl_2_(C_21_H_20_N_2_)]
*M* *_r_* = 477.69
Orthorhombic, 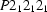

*a* = 7.4807 (6) Å
*b* = 15.1483 (13) Å
*c* = 17.7147 (15) Å
*V* = 2007.4 (3) Å^3^
*Z* = 4
Mo *K*α radiation
μ = 1.20 mm^−1^
*T* = 298 K
0.35 × 0.33 × 0.22 mm

#### Data collection

Bruker SMART 1000 CCD diffractometer
Absorption correction: multi-scan (*SADABS*; Sheldrick, 1996 ▶) *T*_min_ = 0.738, *T*_max_ = 1.000
11233 measured reflections
3956 independent reflections
3871 reflections with *I* > 2σ(*I*)
*R*_int_ = 0.024

#### Refinement

*R*[*F* ^2^ > 2σ(*F* ^2^)] = 0.019
*wR*(*F* ^2^) = 0.052
*S* = 1.01
3956 reflections
238 parameters
H-atom parameters constrained
Δρ_max_ = 0.20 e Å^−3^
Δρ_min_ = −0.52 e Å^−3^
Absolute structure: Flack (1983 ▶), 1670 Friedel pairs
Flack parameter: 0.02 (2)

### 

Data collection: *SMART* (Bruker, 2007 ▶); cell refinement: *SAINT* (Bruker, 2007 ▶); data reduction: *SAINT*; program(s) used to solve structure: *SHELXS97* (Sheldrick, 2008 ▶); program(s) used to refine structure: *SHELXL97* (Sheldrick, 2008 ▶); molecular graphics: *SHELXTL* (Sheldrick, 2008 ▶); software used to prepare material for publication: *SHELXTL*.

## Supplementary Material

Crystal structure: contains datablocks I, global. DOI: 10.1107/S1600536810016466/pk2244sup1.cif

Structure factors: contains datablocks I. DOI: 10.1107/S1600536810016466/pk2244Isup2.hkl

Additional supplementary materials:  crystallographic information; 3D view; checkCIF report

## Figures and Tables

**Fig. 1.**
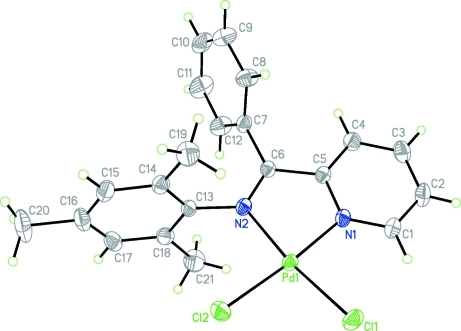
A view of the molecular structure of the title compound with displacement ellipsoids shown at the 30% probability level.

## supplementary crystallographic information

### Comment

Recently, palladium-catalyzed Suzuki-Miyaura reactions involving cross-coupling of aryl halides with aryl boronic acids have emerged as the most important synthetic methods for the preparation of biaryl compounds (Miyaura *et al.*, 1995; Na *et al.*, 2004; Rajagopal *et al.*, 2002; Li 2003; Tomioka *et al.*, 2004; Nicolaou *et al.*, 2005). Thus, because of its utility as an important synthetic methodology, a significant amount of research focus had been devoted to designing improved catalysts for the Suzuki-Miyaura cross-coupling reaction. It is noteworthy that there has been a continuing interest in the further development of more efficient and selective catalytic systems for the synthesis of biaryls. However, only a few examples of *N*,*N' *pyridyl-imine palladium complexes have been reported as catalysts in coupling reaction (Lai *et al.*, 2005; Pelagattia *et al.*, 2005). Herein, we report the synthesis and crystal structure of the title palladium (II) complex that is certainly a potential catalyst in cross-coupling reactions.

The structure of the title compound is a mononuclear configuration with the metal center bound to two N atoms (one from the imine group and one from the pyridine ring) and two Cl atoms (Fig. 1). The coordination geometry around Pd^II^ atom is slightly distorted square planar, and the distances of Pd(1)—N(1) and Pd(1)—N(2) are 2.025 (2) and 2.033 (2) Å, respectively. It is noticed that the *trans* angles (N2—Pd—Cl1 and N1—Pd—Cl2) in the PdN_2_Cl_2_ core do not deviate more than 8° from the ideal value of 180°. Moreover, the planes of the pyridine (N1—C1—C2—C3—C4—C5) and phenyl rings (C8—C9—C10—C11—C12 and C13—C14—C15—C16—C17—C18) are close to perpendicular, and the dihedral angles between them are 80.9 (3) and 82.8 (3)°, respectively. All bond distances and bond angles lie within normal ranges, which are essentially similar to the pyridyl-imine palladium (II) complex (Hsueh *et al.*, 2006; Zhang *et al.*, 2008).

### Experimental

The title compound was prepared by the reaction of palladium(II) dichloride (17.733 mg, 0.1 mmol) with (*E*)-2,4,6-trimethyl-*N*-(phenyl(pyridin-2-yl)methylene)aniline (30.016 mg, 0.1 mmol) in EtOH (20 ml). The mixture was stirred at room temperature for 24 h. The mixture turned yellow immediately. After removal of solvents, dichloromethane (20 ml) was added and the solution was filtered through Celite. The filtrate was slowly evaporated at room temperature to yield yellow crystals suitable for X-ray analysis. Yield: 41.084 mg (86%). Analysis calculated for C_21_H_20_Cl_2_N_2_Pd: C 52.80, H 4.22, N 5.86%; found: C 52.65, H 4.17, N 5.93%.

### Refinement

All H atoms were initially located in a difference Fourier map. The methyl H atoms were then constrained to an ideal geometry with C—H distances of 0.96 Å and *U*_iso_(H) = 1.5*U*_eq_(C), but each group was allowed to rotate freely about its C—C bond. All other H atoms were placed in geometrically idealized positions and constrained to ride on their parent atoms with C—H distances in the range 0.95–1.00 Å and *U*_iso_(H) = 1.2*U*_eq_(C).

### Crystal data

**Table:**

[PdCl_2_(C_21_H_20_N_2_)]	*F*(000) = 960
*M**_r_* = 477.69	*D*_x_ = 1.581 Mg m^−^^3^
Orthorhombic, *P*2_1_2_1_2_1_	Mo *K*α radiation, λ = 0.71073 Å
Hall symbol: P 2ac 2ab	Cell parameters from 4812 reflections
*a* = 7.4807 (6) Å	θ = 2.9–2.6°
*b* = 15.1483 (13) Å	µ = 1.20 mm^−^^1^
*c* = 17.7147 (15) Å	*T* = 298 K
*V* = 2007.4 (3) Å^3^	Parallelpiped, yellow
*Z* = 4	0.35 × 0.33 × 0.22 mm

### Data collection

**Table:**

Bruker SMART 1000 CCD diffractometer	3956 independent reflections
Radiation source: fine-focus sealed tube	3871 reflections with *I* > 2σ(*I*)
graphite	*R*_int_ = 0.024
φ and ω scans	θ_max_ = 26.1°, θ_min_ = 2.7°
Absorption correction: multi-scan (*SADABS*; Sheldrick, 1996)	*h* = −7→9
*T*_min_ = 0.738, *T*_max_ = 1.000	*k* = −16→18
11233 measured reflections	*l* = −21→15

### Refinement

**Table:**

Refinement on *F*^2^	Secondary atom site location: difference Fourier map
Least-squares matrix: full	Hydrogen site location: inferred from neighbouring sites
*R*[*F*^2^ > 2σ(*F*^2^)] = 0.019	H-atom parameters constrained
*wR*(*F*^2^) = 0.052	*w* = 1/[σ^2^(*F*_o_^2^) + (0.040*P*)^2^] where *P* = (*F*_o_^2^ + 2*F*_c_^2^)/3
*S* = 1.01	(Δ/σ)_max_ = 0.002
3956 reflections	Δρ_max_ = 0.20 e Å^−^^3^
238 parameters	Δρ_min_ = −0.52 e Å^−^^3^
0 restraints	Absolute structure: Flack (1983), 1670 Friedel pairs
Primary atom site location: structure-invariant direct methods	Flack parameter: 0.02 (2)

### Special details

**Table:**

Geometry. All esds (except the esd in the dihedral angle between two l.s. planes) are estimated using the full covariance matrix. The cell esds are taken into account individually in the estimation of esds in distances, angles and torsion angles; correlations between esds in cell parameters are only used when they are defined by crystal symmetry. An approximate (isotropic) treatment of cell esds is used for estimating esds involving l.s. planes.
Refinement. Refinement of *F*^2^ against ALL reflections. The weighted *R*-factor wR and goodness of fit *S* are based on *F*^2^, conventional *R*-factors *R* are based on *F*, with *F* set to zero for negative *F*^2^. The threshold expression of *F*^2^ > σ(*F*^2^) is used only for calculating *R*-factors(gt) etc. and is not relevant to the choice of reflections for refinement. *R*-factors based on *F*^2^ are statistically about twice as large as those based on *F*, and *R*- factors based on ALL data will be even larger.

### Fractional atomic coordinates and isotropic or equivalent isotropic displacement parameters (Å^2^)

**Table:**

	*x*	*y*	*z*	*U*_iso_*/*U*_eq_	
Pd1	0.262038 (18)	0.612368 (9)	0.476057 (8)	0.02890 (6)	
Cl1	0.41648 (8)	0.68412 (4)	0.56891 (3)	0.04639 (14)	
Cl2	0.19993 (9)	0.49999 (4)	0.55734 (3)	0.04722 (15)	
N1	0.3065 (2)	0.70552 (11)	0.39625 (10)	0.0321 (4)	
N2	0.1064 (2)	0.56451 (11)	0.39132 (10)	0.0308 (3)	
C1	0.4227 (3)	0.77241 (15)	0.39906 (14)	0.0424 (5)	
H1A	0.4974	0.7776	0.4408	0.051*	
C2	0.4354 (4)	0.83417 (17)	0.34181 (16)	0.0527 (6)	
H2A	0.5191	0.8794	0.3446	0.063*	
C3	0.3231 (4)	0.82774 (16)	0.28102 (14)	0.0498 (6)	
H3A	0.3274	0.8696	0.2426	0.060*	
C4	0.2022 (3)	0.75775 (15)	0.27725 (13)	0.0418 (5)	
H4A	0.1258	0.7518	0.2362	0.050*	
C5	0.1982 (3)	0.69758 (13)	0.33577 (11)	0.0314 (4)	
C6	0.0837 (3)	0.61804 (13)	0.33544 (11)	0.0302 (4)	
C7	−0.0452 (3)	0.60379 (14)	0.27275 (12)	0.0344 (4)	
C8	−0.2010 (4)	0.65290 (18)	0.27039 (14)	0.0493 (6)	
H8A	−0.2234	0.6950	0.3074	0.059*	
C9	−0.3241 (4)	0.6395 (2)	0.21285 (17)	0.0622 (8)	
H9A	−0.4306	0.6712	0.2123	0.075*	
C10	−0.2883 (4)	0.5791 (2)	0.15659 (15)	0.0594 (7)	
H10A	−0.3704	0.5701	0.1179	0.071*	
C11	−0.1303 (5)	0.5321 (2)	0.15769 (16)	0.0674 (8)	
H11A	−0.1052	0.4924	0.1191	0.081*	
C12	−0.0104 (4)	0.54350 (19)	0.21532 (15)	0.0526 (7)	
H12A	0.0948	0.5108	0.2161	0.063*	
C13	0.0126 (3)	0.48204 (13)	0.39711 (12)	0.0316 (4)	
C14	−0.1580 (3)	0.48020 (14)	0.42893 (12)	0.0364 (5)	
C15	−0.2372 (3)	0.39752 (15)	0.43887 (13)	0.0451 (5)	
H15A	−0.3513	0.3946	0.4596	0.054*	
C16	−0.1526 (4)	0.32013 (15)	0.41912 (13)	0.0513 (7)	
C17	0.0171 (4)	0.32429 (15)	0.38896 (14)	0.0469 (6)	
H17A	0.0757	0.2723	0.3761	0.056*	
C18	0.1027 (3)	0.40533 (14)	0.37726 (13)	0.0392 (5)	
C19	−0.2509 (4)	0.56211 (18)	0.45550 (16)	0.0563 (6)	
H19A	−0.3451	0.5464	0.4897	0.084*	
H19B	−0.3004	0.5928	0.4129	0.084*	
H19C	−0.1667	0.5997	0.4808	0.084*	
C20	−0.2428 (7)	0.23101 (19)	0.43118 (19)	0.0839 (11)	
H20A	−0.3672	0.2399	0.4421	0.126*	
H20B	−0.1871	0.2011	0.4727	0.126*	
H20C	−0.2310	0.1959	0.3863	0.126*	
C21	0.2913 (4)	0.40809 (18)	0.34700 (18)	0.0607 (7)	
H21A	0.3515	0.4592	0.3664	0.091*	
H21B	0.2882	0.4109	0.2929	0.091*	
H21C	0.3541	0.3559	0.3625	0.091*	

### Atomic displacement parameters (Å^2^)

**Table:**

	*U*^11^	*U*^22^	*U*^33^	*U*^12^	*U*^13^	*U*^23^
Pd1	0.02800 (8)	0.02850 (8)	0.03020 (9)	0.00077 (6)	−0.00266 (6)	−0.00482 (5)
Cl1	0.0497 (3)	0.0470 (3)	0.0424 (3)	−0.0033 (2)	−0.0130 (3)	−0.0131 (2)
Cl2	0.0583 (4)	0.0409 (3)	0.0424 (3)	−0.0035 (2)	−0.0076 (3)	0.0079 (2)
N1	0.0311 (9)	0.0286 (8)	0.0366 (9)	−0.0025 (7)	0.0017 (7)	−0.0065 (7)
N2	0.0283 (8)	0.0309 (8)	0.0331 (9)	−0.0014 (7)	0.0009 (7)	−0.0058 (7)
C1	0.0406 (12)	0.0392 (12)	0.0472 (13)	−0.0095 (10)	−0.0008 (11)	−0.0067 (10)
C2	0.0574 (16)	0.0424 (13)	0.0583 (15)	−0.0211 (12)	0.0032 (13)	−0.0013 (12)
C3	0.0617 (16)	0.0399 (13)	0.0478 (13)	−0.0088 (12)	0.0065 (12)	0.0057 (11)
C4	0.0473 (14)	0.0419 (12)	0.0360 (11)	−0.0059 (10)	−0.0007 (10)	0.0045 (9)
C5	0.0307 (10)	0.0310 (10)	0.0325 (10)	−0.0008 (8)	0.0022 (8)	−0.0056 (8)
C6	0.0313 (10)	0.0280 (9)	0.0313 (10)	0.0004 (9)	0.0013 (8)	−0.0044 (8)
C7	0.0379 (11)	0.0348 (10)	0.0306 (10)	−0.0073 (9)	−0.0021 (8)	−0.0017 (8)
C8	0.0466 (15)	0.0562 (14)	0.0450 (13)	0.0096 (12)	−0.0083 (11)	−0.0094 (11)
C9	0.0431 (14)	0.079 (2)	0.0646 (18)	0.0041 (13)	−0.0127 (13)	0.0015 (15)
C10	0.0598 (18)	0.0728 (18)	0.0457 (14)	−0.0164 (14)	−0.0216 (13)	−0.0035 (13)
C11	0.076 (2)	0.082 (2)	0.0443 (15)	−0.0021 (17)	−0.0119 (15)	−0.0287 (15)
C12	0.0534 (15)	0.0607 (16)	0.0439 (14)	0.0069 (12)	−0.0049 (11)	−0.0176 (12)
C13	0.0360 (11)	0.0277 (10)	0.0309 (10)	−0.0030 (8)	−0.0043 (8)	−0.0030 (8)
C14	0.0372 (11)	0.0379 (11)	0.0342 (11)	−0.0042 (9)	−0.0007 (9)	−0.0016 (9)
C15	0.0440 (12)	0.0521 (13)	0.0392 (11)	−0.0149 (12)	−0.0013 (10)	0.0018 (9)
C16	0.081 (2)	0.0354 (12)	0.0378 (12)	−0.0186 (12)	−0.0085 (12)	0.0033 (10)
C17	0.0682 (17)	0.0292 (11)	0.0433 (13)	−0.0007 (11)	−0.0094 (12)	−0.0043 (10)
C18	0.0442 (12)	0.0351 (11)	0.0383 (12)	0.0021 (9)	−0.0060 (9)	−0.0054 (9)
C19	0.0447 (13)	0.0544 (14)	0.0696 (15)	0.0053 (12)	0.0157 (13)	−0.0040 (12)
C20	0.130 (3)	0.0479 (15)	0.0738 (19)	−0.040 (2)	0.011 (3)	0.0052 (14)
C21	0.0497 (15)	0.0511 (15)	0.0814 (19)	0.0127 (12)	0.0105 (14)	−0.0107 (14)

### Geometric parameters (Å, °)

**Table:**

Pd1—N1	2.0250 (18)	C10—H10A	0.9300
Pd1—N2	2.0334 (17)	C11—C12	1.370 (4)
Pd1—Cl2	2.2776 (6)	C11—H11A	0.9300
Pd1—Cl1	2.2851 (5)	C12—H12A	0.9300
N1—C1	1.336 (3)	C13—C18	1.389 (3)
N1—C5	1.348 (3)	C13—C14	1.396 (3)
N2—C6	1.291 (3)	C14—C15	1.397 (3)
N2—C13	1.436 (3)	C14—C19	1.498 (3)
C1—C2	1.383 (4)	C15—C16	1.377 (4)
C1—H1A	0.9300	C15—H15A	0.9300
C2—C3	1.369 (4)	C16—C17	1.378 (4)
C2—H2A	0.9300	C16—C20	1.525 (3)
C3—C4	1.395 (3)	C17—C18	1.400 (3)
C3—H3A	0.9300	C17—H17A	0.9300
C4—C5	1.381 (3)	C18—C21	1.509 (4)
C4—H4A	0.9300	C19—H19A	0.9600
C5—C6	1.479 (3)	C19—H19B	0.9600
C6—C7	1.486 (3)	C19—H19C	0.9600
C7—C8	1.384 (3)	C20—H20A	0.9600
C7—C12	1.392 (3)	C20—H20B	0.9600
C8—C9	1.389 (4)	C20—H20C	0.9600
C8—H8A	0.9300	C21—H21A	0.9600
C9—C10	1.379 (4)	C21—H21B	0.9600
C9—H9A	0.9300	C21—H21C	0.9600
C10—C11	1.380 (5)		
			
N1—Pd1—N2	80.04 (7)	C10—C11—C12	120.4 (3)
N1—Pd1—Cl2	174.72 (5)	C10—C11—H11A	119.8
N2—Pd1—Cl2	94.79 (5)	C12—C11—H11A	119.8
N1—Pd1—Cl1	95.05 (5)	C11—C12—C7	120.3 (3)
N2—Pd1—Cl1	172.09 (5)	C11—C12—H12A	119.8
Cl2—Pd1—Cl1	90.21 (2)	C7—C12—H12A	119.8
C1—N1—C5	119.2 (2)	C18—C13—C14	122.0 (2)
C1—N1—Pd1	127.55 (16)	C18—C13—N2	118.19 (19)
C5—N1—Pd1	113.20 (13)	C14—C13—N2	119.53 (18)
C6—N2—C13	122.46 (17)	C15—C14—C13	117.2 (2)
C6—N2—Pd1	114.68 (13)	C15—C14—C19	120.4 (2)
C13—N2—Pd1	122.48 (13)	C13—C14—C19	122.3 (2)
N1—C1—C2	122.0 (2)	C16—C15—C14	122.4 (2)
N1—C1—H1A	119.0	C16—C15—H15A	118.8
C2—C1—H1A	119.0	C14—C15—H15A	118.8
C3—C2—C1	119.1 (2)	C15—C16—C17	118.9 (2)
C3—C2—H2A	120.5	C15—C16—C20	121.0 (3)
C1—C2—H2A	120.5	C17—C16—C20	120.2 (3)
C2—C3—C4	119.3 (2)	C18—C17—C16	121.3 (2)
C2—C3—H3A	120.3	C18—C17—H17A	119.4
C4—C3—H3A	120.3	C16—C17—H17A	119.4
C5—C4—C3	118.7 (2)	C13—C18—C17	118.3 (2)
C5—C4—H4A	120.7	C13—C18—C21	121.4 (2)
C3—C4—H4A	120.7	C17—C18—C21	120.3 (2)
N1—C5—C4	121.65 (19)	C14—C19—H19A	109.5
N1—C5—C6	115.08 (18)	C14—C19—H19B	109.5
C4—C5—C6	123.19 (19)	H19A—C19—H19B	109.5
N2—C6—C5	115.63 (17)	C14—C19—H19C	109.5
N2—C6—C7	124.55 (18)	H19A—C19—H19C	109.5
C5—C6—C7	119.82 (17)	H19B—C19—H19C	109.5
C8—C7—C12	119.2 (2)	C16—C20—H20A	109.5
C8—C7—C6	119.40 (19)	C16—C20—H20B	109.5
C12—C7—C6	121.3 (2)	H20A—C20—H20B	109.5
C7—C8—C9	120.1 (2)	C16—C20—H20C	109.5
C7—C8—H8A	119.9	H20A—C20—H20C	109.5
C9—C8—H8A	119.9	H20B—C20—H20C	109.5
C8—C9—C10	119.9 (3)	C18—C21—H21A	109.5
C8—C9—H9A	120.0	C18—C21—H21B	109.5
C10—C9—H9A	120.0	H21A—C21—H21B	109.5
C11—C10—C9	119.9 (2)	C18—C21—H21C	109.5
C11—C10—H10A	120.0	H21A—C21—H21C	109.5
C9—C10—H10A	120.0	H21B—C21—H21C	109.5
